# Allergy-related outcomes and sleep-related disorders in adults: a cross-sectional study based on NHANES 2005–2006

**DOI:** 10.1186/s13223-022-00669-z

**Published:** 2022-03-22

**Authors:** Yang Xi, Yu-Qin Deng, Shi-Ming Chen, Yong-Gang Kong, Yu Xu, Fen Li, Wo-Er Jiao, Gan Lu, Ze-Zhang Tao

**Affiliations:** 1 Department of Otolaryngology, Head and Neck Surgery, Renmin Hospital of Wuhan University, 238 Jie-Fang Road, Wuhan, 430060 Hubei People’s Republic of China; 2 Institute of Otolaryngology, Head and Neck Surgery, Renmin Hospital of Wuhan University, Wuhan, People’s Republic of China

**Keywords:** Allergy, Sleep disorders, Epidemiology, Clinical study

## Abstract

**Background:**

Epidemiological evidence between the sleep disorders and allergy-related outcomes is limited.

**Objectives:**

The purpose of the present study was to estimate the relationship between sleep disorders and allergy-related outcomes in adults.

**Methods:**

We built logistic regression models to examine the associations between sleep disorders and allergy-related outcomes in adult participants using the 2005–2006 NHANES database. Allergy-related outcomes included sIgE levels, asthma, hay fever, sneezing, wheezing, and eczema. Sleep disorders included sleep latency, sleep length, sleep problems, OSA symptoms, and daytime sleepiness. A t-test was used for between-group comparisons.

**Results:**

Participants with OSA symptoms had 2.72 × higher odds of experiencing hay fever and 1.54 × higher odds of having eczema compared to Non-OSA symptoms participants. Participants with insufficient sleep (≤ 6 h/night) had 1.27 × higher odds of developing allergic sensitisation compared to participants with adequate sleep (7–8 h/night). Sneezing was positively associated with sleep problems (OR: 1.706; 95% CI 1.386, 2.099), OSA symptoms (OR: 1.297; 95% CI 1.049, 1.605), and daytime sleepiness (OR: 1.569; 95% CI 1.205, 2.04).

**Conclusion:**

Our findings suggest a positive association between allergy-related outcomes and sleep disorders. In particular, OSA symptoms, daytime sleepiness, and sleep problems are strongly associated with allergic conditions.

**Graphical Abstract:**

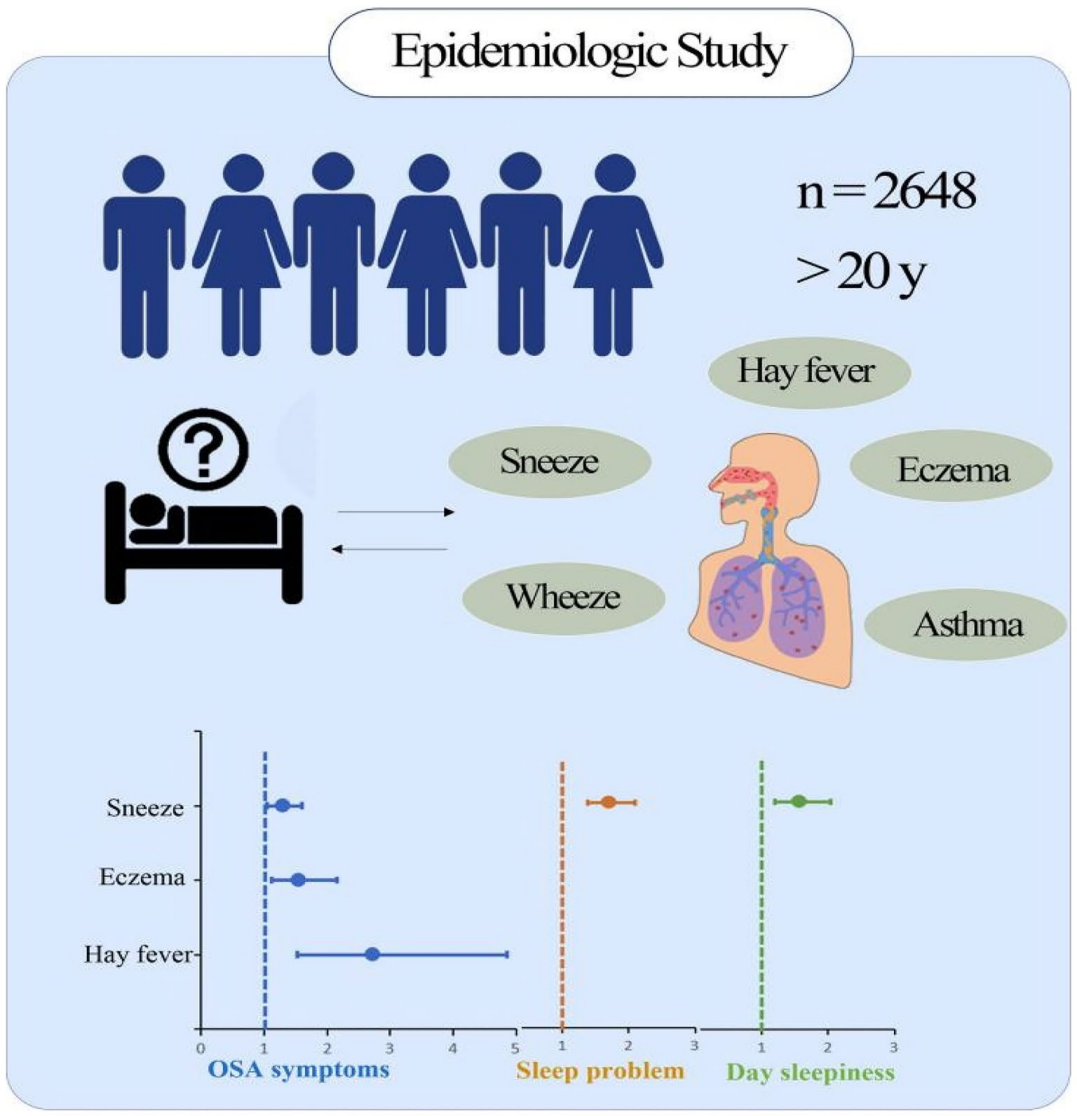

## Introduction

Allergic disease is highly prevalent and has become a major public health problem worldwide [1]. Over the past several decades, IgE sensitisation has increased significantly and is a key factor in the development of allergic diseases [2]. Ordinary allergic diseases include eczema, asthma, allergic rhinitis, and hay fever. Eczema typically occurs at a young age and may develop into wheezing and allergic rhinitis. Persistent wheezing may develop into asthma [3], which affects approximately 235 million people worldwide [4]. The annual direct and indirect costs of allergic rhinitis reached USD 24.8 billion in 2014 [5]. Recent surveys have indicated that the global incidence of allergic reactions is between 50 and 112 cases per 100,000 individuals per year, with a lifetime prevalence of approximately 0.3–5.1% [6]. These data underscore the heavy burden imposed by allergic diseases on societal health and the economy. Therefore, identifying effective preventive methods and modifiable risk factors for allergic rhinitis is critical.

Sleep disorders are a large category of sleep-related disorders, including insufficient sleep time, difficulty falling asleep, early awakening, poor sleep quality, circadian rhythm disorders, insomnia, and breathing-related disorders [7]. Sleep disorders can result in daytime sleepiness [8]. Insufficient sleep is a causative factor for various chronic diseases, including obesity, diabetes, and cardiovascular disease (CVD), which are often co-morbid with sleep disorders [9]. Further, demographic characteristics such as smoking, drinking, obesity, and household income, in addition to genetic factors, modulate the risk of sleep disorders and allergies.

Growing evidence supports an association between sleep disorders and allergy-related outcomes. In patients with seasonal allergies, issues such as fatigue and sleep disorders in addition to disease-specific symptoms are exacerbated during the pollen season [10]. This may be related to nasal congestion caused by allergic rhinitis. Mechanical obstruction caused by nasal congestion may lead to sleep apnoea [11]. A recent study of more than 5000 patients from 10 European countries reported that insufficient sleep time (< 6 h) was associated with respiratory and nasal symptoms [12]. The interaction between sleep and allergic skin diseases is considered clinically significant, and sleep quality can positively affect the progress of allergic skin diseases [13]. Obstructive sleep apnea (OSA) is the most common sleep disorder. AR may increase airway resistance due to higher nasal resistance, causing symptoms such as apnea and snoring, thereby promoting the occurrence and development of OSA [14]. The increased levels of cytokines interleukin (IL)-1β, IL-4 and IL-10 in AR patients have been shown to be related to the increase of latency of rapid eye movement (REM) sleep and the shortening of REM sleep time [15], and worsen the sleep quality of OSA patients [16]. However, some studies have shown that AR is not the main risk factor for OSA [17].

These findings collectively suggest various degrees of an association between sleep disorders and allergic diseases of respiratory tract, nose, and skin. Nevertheless, there is a paucity of studies on this potential association and previous studies have been limited by small sample sizes. Here, we analyse data from the National Health and Nutrition Examination Survey (NHANES) database to examine the relationship between sleep and allergies in adults older than 20 years old among the large population of the US.

## Methods

### Epidemiologic study population

The present study used data from the National Health and Nutrition Examination Survey (NHANES) 2005–2006, a cross-sectional study that evaluated the nutrition and health of adults and children in the US. NHANES is a major program of the National Center for Health Statistics (NCHS). The survey used a complex multi-stage design, and the sample was selected to represent the population of all age groups in the US. The Research Ethics Review Board at the NCHS approved the survey protocol [18].

NHANES 2005–2006 is the only cycle of complete questionnaires on sleep habits and allergy-related results. In total, 10,348 participants were investigated in the NHANES from 2005 to 2006. Our analysis was restricted to 2648 individuals with complete information on allergies, sleep disorders, and model covariates.

### Assessment of allergy-related results

This study assessed self-reported current allergy conditions and allergic sensitisation as measured by specific immunoglobulin E(sIgE). Data about allergy conditions was received from a questionnaire that was completed during the NHANES clinic visit. Participants under the age of 16 years old were interviewed by a proxy interviewee (typically their parents). Allergen sIgE levels in serum samples were measured using the Pharmacia Diagnostics ImmunoCAP 1000 System (Kalamazoo, Michigan). Individuals who tested positive for at least one allergen (≥ 0.35 kU/L) were considered allergen-sensitised (sIgE-positive) [19]. The five following allergic symptoms were identified from the questionnaire [20, 21]:

- Hay fever: Have you had hay fever in the past year? (AGQ030)
- Sneezing: In the past year, did you have any sneezing, runny nose? (AGQ100)
- Asthma: In the past year, have you had a history of acute asthma attacks? (MCQ040)
- Wheezing: In the past year, have you had wheezing? (RDQ070)
- Eczema: Have your doctors or health professionals informed you that you have eczema? (AGQ180)

### Assessment of sleep disturbances

We investigated sleep disorder outcomes, including sleep duration, sleep onset latency, OSA symptoms, sleep problems, and daytime sleepiness using a self-questionnaire. Outcomes were defined as follows [9]:

- Sleep duration [22] was classified as ≤ 6 h/night, 7–8 h/night, or ≥ 9 h/night. (SLD010H).
- Sleep onset latency [23] was categorised into 6–30 min/night, > 30 min/night, or ≤ 5 min/night. (SLD020M).

OSA symptoms [9, 24]:

- Have you ever been informed you that you have a sleep disorder? (SLQ070A);
- Snoring three nights or more per week while sleeping (SLQ030);
- Snorting, gasping, or cessation of breathing for three nights or more every week during sleep (SLQ040);
- Feeling extremely sleepy during the daytime 16–30 times a month. (SLQ120).
- The presence of one or more of the above issues was defined as the presence of OSA symptoms.

Sleep problems [9]:

- Have you ever been informed you that you have trouble sleeping? (SLQ050);
- Have you ever been informed you that you have a sleep disorder? (SLQ060);
- How often did you have difficulty in falling asleep in the past month? (SLQ080);
- How often did you wake up during the night and were unable to get back to sleep in the past month? (SLQ090);
- How often did you wake up too early in the morning and have trouble in getting back to sleep in the past month? (SLQ100)
- Responses of "frequently" or more (≥ 5 times/month) to any of the above questions were considered to indicate the presence of sleep problems.

Daytime sleepiness [9]:

- How often did you feel sleepy during the day, even though you have enough sleep at night in the past month? (SLQ110);
- How often did you wake up too early in the morning and were unable to get back to sleep in the past month?
- Responses of "frequently" or more (≥ 5 times/month) to any of the above questions were considered to indicate the presence of daytime sleepiness.

### Covariates

We adjusted the regression model for allergy-related covariates. Demographic information including age, gender, race/ethnicity (Mexican American, other Hispanics, non-Hispanic white, non-Hispanic black, and other races), education level (less than high school, completed high school, and more than high school), and household poverty income ratio (PIR, ≤ 1, > 1) was obtained via questionnaires [25]. Participants' weight divided by height squared (kg/m^2^) was the body mass index (BMI). Participants were classified into normal weight (BMI < 25 kg/m^2^), overweight (BMI of 25–29 kg/m^2^), or obesity (≥ 30 kg/m^2^). Drinking and smoking information was obtained via questionnaires. Drinking alcohol was classified as drinking 1–4 glasses of wine a week or drinking more than 4 glasses of wine a week. Smoking was divided into current smoker, never smoked (smoked < 100 cigarettes), or former smoker (not a current smoker but had smoked ≥ 100 cigarettes) [9].

In addition to the above model, this study also added CVD and diabetes that have an impact on sleep disorders [26, 27]. CVD was defined as participants with previous congestive heart failure, coronary heart disease, angina pectoris, heart disease, and hypertension, which can be obtained from the medical health questionnaire. The logistic regression model also adjusted the physical and mental health status in the self-report.

### Statistical analysis

Demographics, lifestyle, and questionnaire results were expressed as weighted percentages. The Rao-Scott χ^2^ test was used to compare the percentages of categorical variables between participants with and without allergic sensitisation [29]. A logistic regression model was established to examine the relationship between sleep disorders and allergy-related outcomes. Multiple linear regression analysis was performed using the Enter method. Gender, age, race/ethnicity, education level, poverty income ratio (PIR), BMI, smoking status, and alcohol consumption were adjusted in Model 1. Model 2 was adjusted for the covariates of Model 1, diabetes, CVD, and physical/mental health.

The sampling weight of the population sampling examination (WTMEC2YR) and study design variables (SDMVPSU and SDMVSTRA) were used for data analysis. In all analyses, the main sampling unit (SDMVPSU), strata (SDMVSTRA), and weight were specified using the complex sample module in SPSS, taking into account the complexity of the sampling design. For analysis of clinical data, a t-test was used to compare mean differences in variables between allergy and control groups.

## Results

### Study population for epidemiologic analysis

Sample sizes and weighting characteristics of the study sample are listed in Table 1. Study sample size was 2648 as a representation of the US population of adults aged 20 years and older. Of participants, 47.9% were women, and 90.6% lived above the PIR. The survey population predominantly comprised non-Hispanic whites, accounting for 77.2% of the population. Of participants, 62.5%, 46.4%, and 14.8% had attended college, had never smoked, and drank more than four glasses of wine per week, respectively. Most participants did not have CVD (71.3%) or diabetes (94.2%). Additional baseline characteristics of participants stratified by specific IgE sensitisation are summarised in Table 1. In addition to age (P < 0.001) and gender (P < 0.001), specific IgE sensitisation was associated with race/ethnicity (P < 0.001), PIR (P = 0.009), and smoking status (P = 0.001). No significant differences were observed in specific IgE sensitisation by BMI (P = 0.988), alcohol consumption (P = 0.762), education level (P = 0.251), diabetes (P = 0.695), or CVD (P = 0.427).

**Table 1:** Sample size (n) and weighted characteristics of NHANES 2005–2006 participants

Characteristics	Total		Sensitized		Nonsensitized		P value
	N	Weighted	N	Weighted	N	Weighted	
Sex							**< 0.001**
Male, % (SE)	1413	52.1 (0.9)	745	51.3 (1.3)	668	48.7 (1.3)	
Female, % (SE)	1235	47.9(0.9)	505	39.4(1.5)	730	60.6 (1.5)	
Age (years)							**< 0.001**
18–44, % (SE)	1364	51.2 (2.3)	726	52.5 (1.7)	638	47.5 (1.7)	
45–69, % (SE)	959	40.5 (1.7)	410	40.0 (1.1)	549	60.0 (1.1)	
≥ 70, % (SE)	325	8.4 (1.1)	114	30.4 (2.5)	211	69.6 (2.5)	
Race/Ethnicity							** < 0.001**
Mexican American, % (SE)	507	7.4 (1.0)	236	47.2 (2.0)	271	52.8 (2.0)	
Other Hispanic, % (SE)	73	2.8 (0.8)	43	58.8 (6.2)	30	41.2 (6.2)	
Non-Hispanic White, % (SE)	1496	77.2 (2.4)	637	43.2 (1.3)	859	56.8 (1.3)	
Non-Hispanic Black, % (SE)	479	8.3 (1.4)	288	59.7 (3.3)	191	40.3 (3.3)	
Other race—including multi-racial, % (SE)	93	4.2 (0.5)	46	49.2 (4.8)	47	50.8 (4.8)	
BMI							0.988
Normal, % (SE)	821	33.9 (1.7)	380	45.5 (1.7)	441	54.5 (1.7)	
Overweight, % (SE)	930	33.3 (1.3)	434	45.6 (1.3)	496	54.4 (1.3)	
Obese, % (SE)	876	32.8 (1.7)	427	45.5 (2.2)	449	54.4 (2.2)	
PIR							**0.009**
≤ 1 (under poverty level), % (SE)	370	9.4 (0.7)	202	55.8 (3.6)	168	44.2 (3.6)	
> 1 (above poverty level), % (SE)	2184	90.6 (0.7)	1005	44.6 (1.2)	1179	55.4 (1.2)	
Smoking status							**0.001**
Never smokers, % (SE)	1234	46.4 (1.1)	626	50.1 (1.8)	608	49.9 (1.8)	
Former smokers, % (SE)	838	30.7 (1.3)	367	42.4 (2.0)	471	57.6 (2.0)	
Current smokers, % (SE)	576	22.9 (1.4)	257	40.6 (1.5)	319	59.4 (1.5)	
Alcohol consumption							0.762
1–4 drinks per week, % (SE)	2236	85.2 (0.9)	1051	45.7 (1.3)	1185	54.3 (1.3)	
> 4 drinks per week, % (SE)	412	14.8 (0.7)	199	44.7 (2.9)	213	55.3 (2.9)	
Education level							0.251
Less than High School, % (SE)	580	13.8 (1.3)	269	44.0 (2.6)	311	56.0 (2.6)	
Completed High School, % (SE)	628	23.7 (0.8)	284	42.5 (2.0)	344	57.5 (2.0)	
More than High School, % (SE)	1440	62.5 (1.9)	697	47.0 (1.8)	743	53.0 (1.8)	
Diabetes							0.695
Yes, % (SE)	193	5.8 (0.6)	87	47.9 (5.7)	106	52.1 (5.7)	
No, % (SE)	2409	94.2 (0.6)	1147	45.6 (1.1)	1262	54.4 (1.1)	
CVD							0.427
Yes, % (SE)	789	28.7 (1.1)	361	44.1 (2.3)	428	55.9 (2.3)	
No, % (SE)	1856	71.3 (1.1)	887	46.1 (1.2)	969	53.9 (1.2)	

Sensitised = serum‐specific IgE ≥ 0.35 kUA/L; Nonsensitised = serum‐specific IgE < 0.35 kUA/L. Proportions were compared using the Rao-Scott χ^2^ test

### Sleep disorders in the study population

Of participants, 34.6% slept less than 7 h per night, whereas only 6.3% of participants slept more than 9 h a night (Table 2). 31.2% of the participants fell asleep within 5 min every night, which may also be a sign of extreme lack of sleep. In contrast, 14.7% of participants fell asleep more than 30 min. 42.2% of the participants reported having sleep problems, including difficulty falling asleep and waking up at night or early in the morning. Of participants, 27% reported feeling excessive sleepiness during the day and 51.8% presented with OSA symptoms.

**Table 2:** Sleep disorders of NHANES 2005–2006 participants

Characteristics	Total		Male		Female		P value
	N	Weighted	N	Weighted	N	Weighted	
Sleep duration							0.26
≤ 6 h/night, %	965	34.6 (1.2)	535	55.6 (2.1)	430	44.4 (2.1)	
7–8 h/night, %	1488	59.0 (1.2)	790	51.3 (1.2)	698	48.7 (1.2)	
≥ 9 h/night, %	192	6.3 (0.4)	88	40.9 (4.4)	104	59.1 (4.4)	
Sleep onset latency time							0.32
≤ 5 min, %	781	31.2 (1.7)	455	56.9 (2.2)	326	43.1 (2.2)	
5–30 min, %	1419	54.0 (1.7)	738	50.6 (1.4)	681	49.4 (1.4)	
> 30 min, %	436	14.7 (0.8)	215	47.7 (2.0)	221	52.3 (2.0)	
Sleep problems							** < 0.001**
Yes, %	1072	42.2 (0.9)	513	46.9 (1.4)	559	53.1 (1.4)	
No, %	1576	57.8 (0.9)	900	55.9 (1.4)	676	44.1 (1.4)	
OSA symptoms							**0.001**
Yes, %	1323	51.8 (1.5)	800	60.1 (1.5)	523	39.9 (1.5)	
No, %	1325	48.2 (1.5)	613	43.5 (1.3)	712	56.5 (1.3)	
Day sleepiness							**< 0.001**
Yes, %	710	27.3 (1.0)	303	43.5 (1.8)	407	56.5 (1.8)	
No, %	1938	72.7 (1.0)	1110	55.3 (1.2)	828	44.7 (1.2)	

OSA: obstructive sleep apnoea

### Associations between sleep disorders and allergic sensitisation

**Fig. 1:**
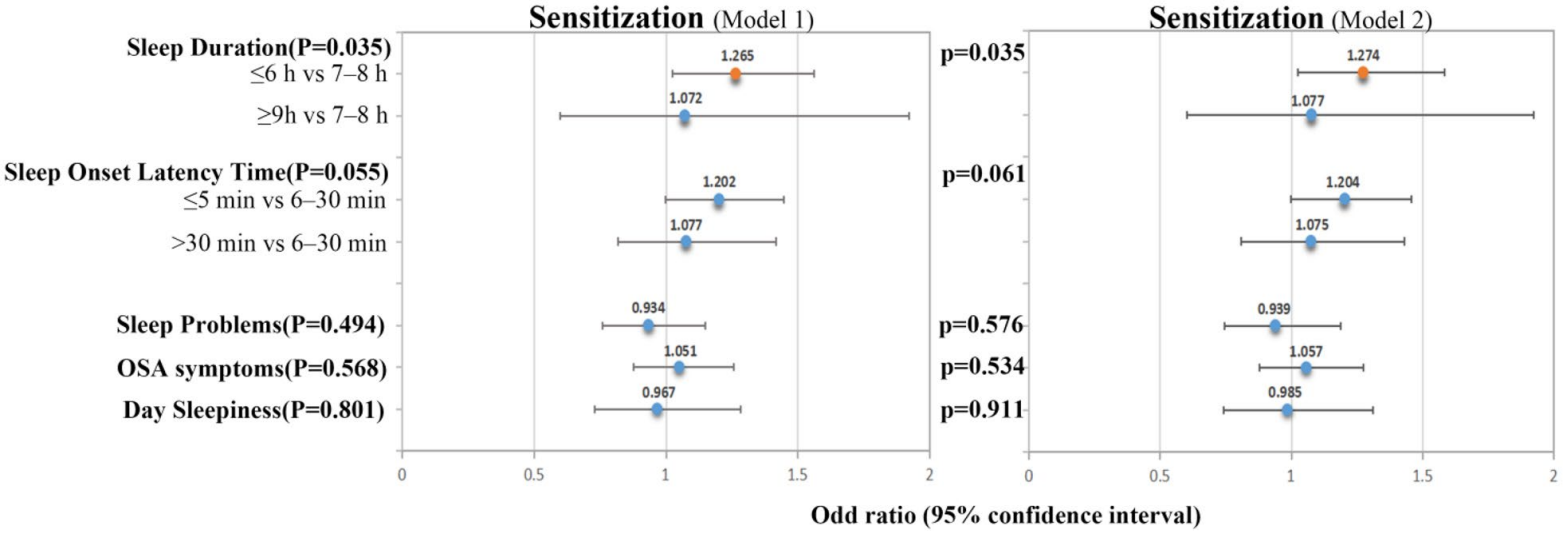
Associations (OR [95% CI]) between sleep disorders and allergic sensitisation in NHANES 2005–2006 participants. Model 1 was adjusted for gender, age, race/ethnicity, education level, PIR, BMI, smoking status, and alcohol consumption. Model 2 was adjusted for Model 1, diabetes, CVD, and physical/mental health. Orange dots indicate statistical significance (p-value < 0.05)

The calibration model of logistic regression analysis revealed that participants with insufficient sleep (≤ 6 h/night) were more likely to develop allergic sensitisation compared to participants with adequate sleep (7–8 h/night) (OR: 1.265; 95% CI 1.025, 1.562). The relationship was not altered after further adjustment in Model 2 (OR: 1.274; 95% CI 1.024, 1.584). No significant associations were noted was between sleep latency, sleep problems, OSA symptoms, daytime sleepiness, and allergic sensitisation (Fig. 1).

### Associations between sleep disorders and allergic symptoms

**Fig. 2:**
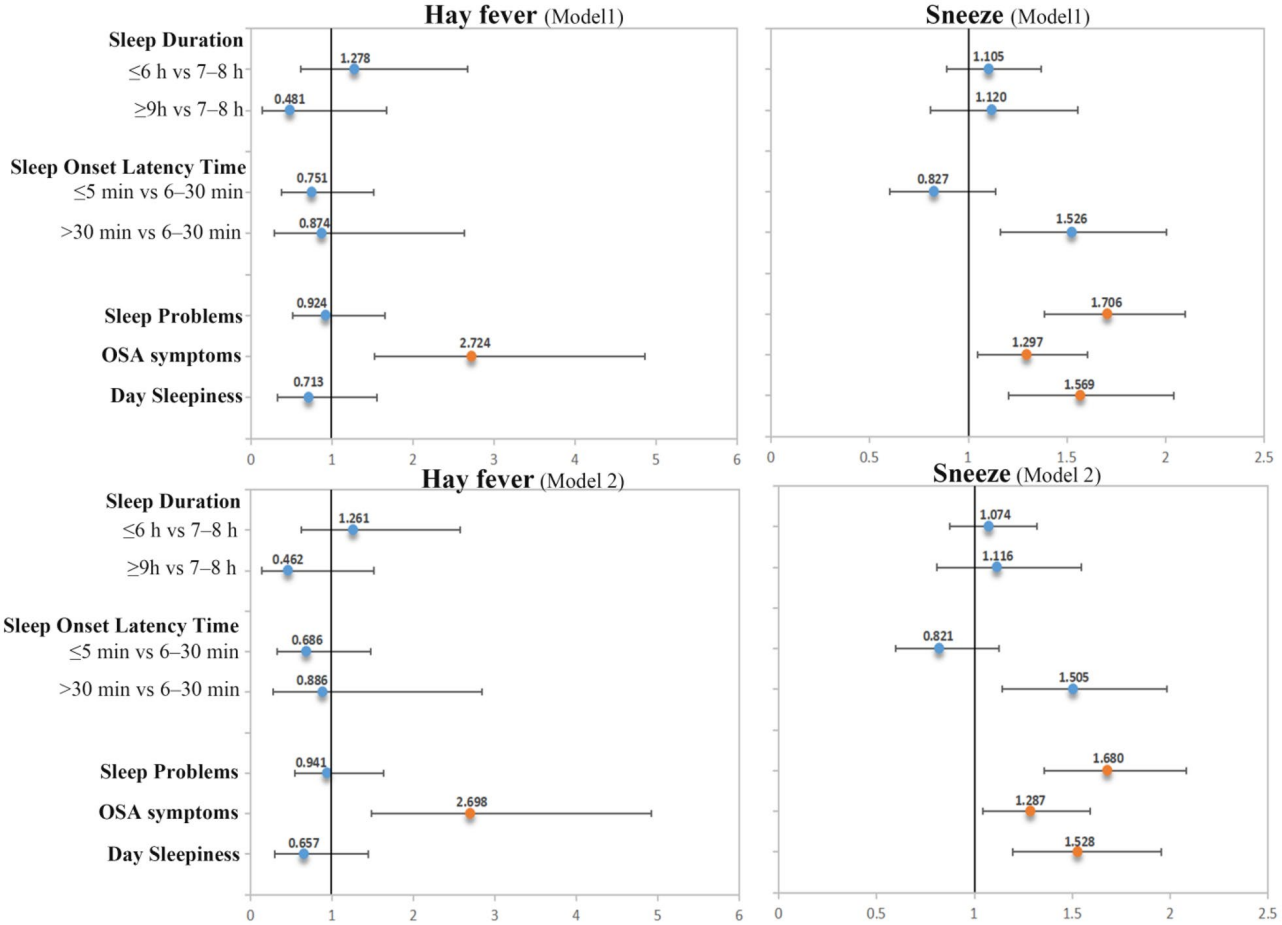
Associations (OR [95% CI]) between sleep disorders and allergic symptoms (hay fever and sneezing) in NHANES 2005–2006 participants. Model 1 was adjusted for gender, age, race/ethnicity, education level, PIR, BMI, smoking status, and alcohol consumption. Model 2 was adjusted for Model 1, diabetes, CVD, and physical/mental health. Orange dots indicate statistical significance (p-value < 0.05)

Associations between sleep disorders and hay fever, sneezing are presented in Fig. 2 Participants with OSA symptoms were 2.72 × more likely to have hay fever compared to the Non-OSA symptoms population (OR: 2.724; 95% CI 1.526, 4.861). The direction of the association did not change after adjusting for covariates (OR: 2.698; 95% CI 1.480, 4.916). Significant positive associations were noted between sneezing symptoms and sleep disorders, including sleep problems (Model 1: 1.706 [1.386, 2.099]; Model 2: 1.680 [1.356, 2.080]), OSA symptoms (Model 1: 1.297 [1.049, 1.605]; Model 2: 1.287 [1.042, 1.589]), and daytime sleepiness (Model 1: 1.569 [1.205, 2.043]; Model 2: 1.528 [1.194, 1.955]).

**Fig. 3:**
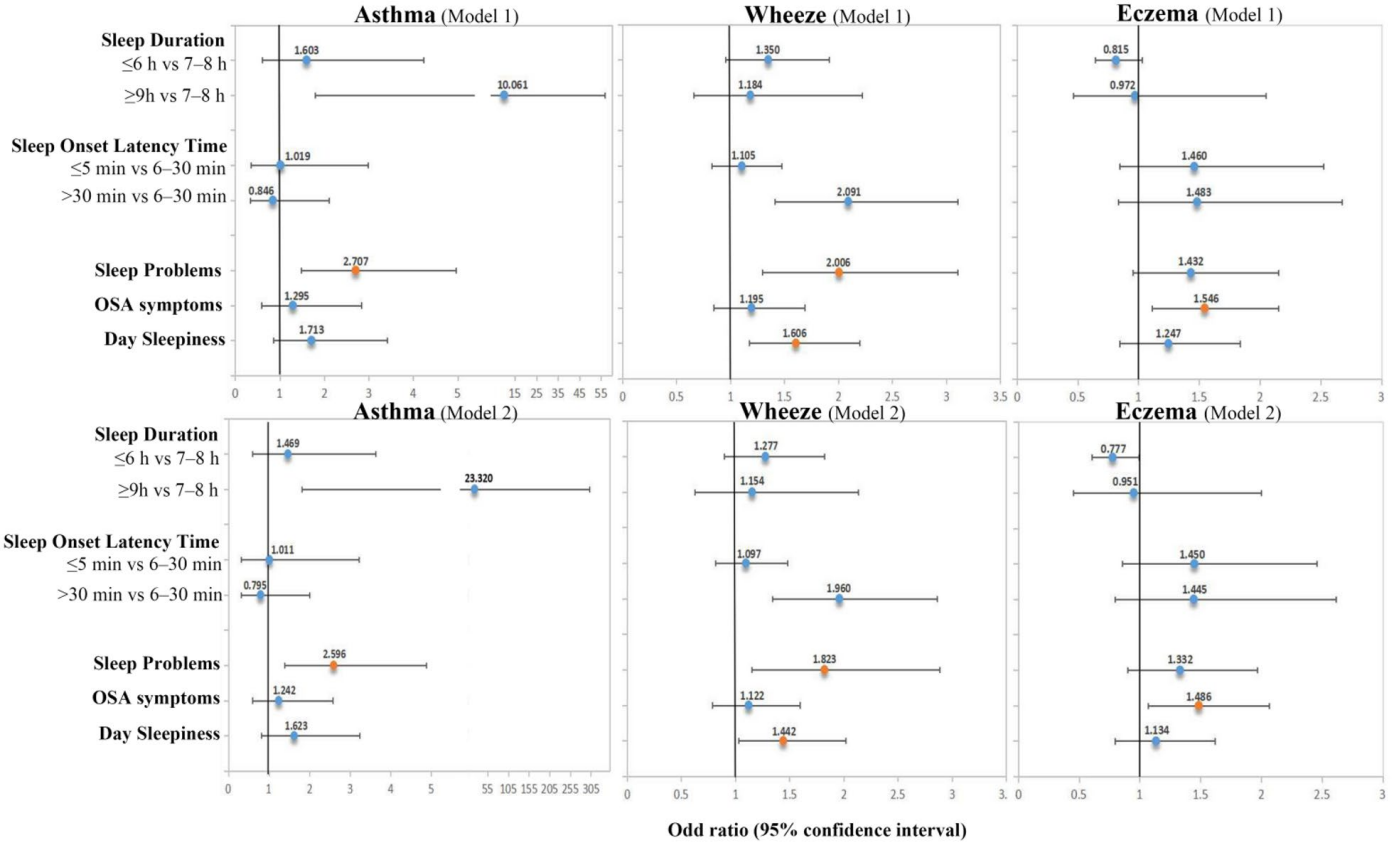
Associations (OR [95% CI]) between sleep disorders and allergic symptoms (asthma, wheezing, and eczema) in NHANES 2005–2006 participants. Model 1 was adjusted for gender, age, race/ethnicity, education level, PIR, BMI, smoking status, and alcohol consumption. Model 2 was adjusted for Model 1, diabetes, CVD, and physical/mental health. Orange dots indicate statistical significance (p-value < 0.05)

Relationships between sleep disorders and asthma, wheezing, and eczema are presented in Fig. 3. A positive correlation was noted between asthma symptoms and OSA symptoms (Model 1: 2.707 [1.477, 4.961]; Model 2: 2.596 [1.381, 4.879]). Wheezing symptoms were positively correlated with sleep problems and daytime sleepiness. Individuals with sleep problems were twice as likely to develop wheezing symptoms compared to the healthy population (Model 1: 2.006 [1.297, 3.102]; Model 2: 1.823 [1.151, 2.885]). Individuals with daytime sleepiness were 60% more likely to have wheezing symptoms compared to healthy individuals (Model 1: 1.606 [1.176, 2.194]; Model 2: 1.442 [1.031, 2.017]). Individuals with symptoms of sleep apnoea were 54.6% more likely to develop eczema compared to the Non-OSA symptoms population (Model 1: 1.546 [1.112, 2.150], Model 2: 1.486 [1.069, 2.066]).

## Discussion

This study examined the relationship between allergic outcomes and sleep disorders through an epidemiological research. We observed that allergy-related outcomes may be positively correlated with sleep disorders. Based on 2005–2006 NHANES data of 2,648 participants over the age of 20, our logistic regression model revealed that participants with OSA symptoms had 2.72 × higher odds of hay fever and 1.54 × higher odds of eczema compared to the Non-OSA symptoms population. Participants with insufficient sleep (≤ 6 h/night) are more likely to have elevated sIgE levels than participants with adequate sleep (7–8 h/night). Sneezing symptoms were positively associated with sleep problems, OSA symptoms, and daytime sleepiness. These findings suggest that allergies are positively associated with sleep disorders. In particular, sleep duration is positively correlated with sIgE sensitisation. OSA symptoms, daytime sleepiness, and sleep problems were positively associated with various self-reported allergic conditions.

There is a paucity of epidemiological studies on the relationship between allergies and sleep disorders. Here, we analysed data on 2648 NHANES participants aged 20 years or older and considered age, sex, race/ethnicity, socioeconomic status, and smoking status. Given that underlying diseases and daily alcohol consumption may affect sleep quality [27, 28, 30], we adjusted for confounding factors such as alcohol consumption and underlying diseases including diabetes and CVD. Therefore, in our research, we subdivided the allergy-related results into sIgE sensitisation and self-reported allergic conditions(including hay fever, sneezing, asthma, wheezing, and eczema), and performed a logical regression analysis between them and sleep duration, sleep latency, sleep problems, obstructive sleep apnea, and daytime sleepiness one by one, in order to find a more accurate relationship.

A study by Luyster et al. based on 2007–2012 NHANES data reported that adults over the age of 20 years old who had a short sleep time experienced an increase in asthma attacks [31]. A randomised controlled study [32] reported that sleep deprivation significantly reduced the response threshold for IgE-induced peanut allergy. An observational cross-sectional study [33] reported that adults with atopic dermatitis were more likely to have sleep disorders, including shorter sleep times, difficulties falling asleep and awakening early in the morning. These studies have shown a link between sleep duration and allergic diseases. The length of sleep can affect the immune function by affecting the innate immune system or cytokines [34]. Although the specific mechanism remains to be further studied, it is believed that lack of sleep may increase the number of B lymphocytes and the level of immunoglobulin [35, 36], promote the release of inflammatory factors, and thus participate in IgE-mediated allergic diseases. This is similar to the findings of this study. Participants with insufficient sleep (≤ 6 h/night) are more likely to have allergic sensitisation than participants with adequate sleep (7–8 h/night).

The positive correlation mechanism between certain allergic symptoms and sleep disorders warrants further exploration. Allergy patients typically experience sleep-disordered breathing, nasal mucosa oedema, and nasal congestion caused by allergic rhinitis [37]. The nose accounts for half of the resistance of the entire respiratory system. Nasal congestion can cause changes in airflow speed and resistance, which affects the pressure difference between the atmosphere and chest cavity. During inhalation, the negative pressure in the chest pulls the soft tissues of the upper airway closer, resulting in partial or complete obstruction [38]. This phenomenon may cause nasal mucosal oedema, leading to sleep snoring and apnoea at night [37]. This view supports our findings of a positive correlation between sleep apnoea, hay fever, and sneezing symptoms.

Daytime sleepiness is a crucial symptom of OSA symptoms [39] and may be similarly associated with allergies. In this regard, participants with sleep apnoea were 1.54 × more likely to develop eczema compared to healthy individuals. This agrees with the findings of Camfferman et al. [40], who reported that sleep disorders affect patients with eczema. They proposed that eczema-associated itching is caused by neuropeptide-mediated vasodilation, and subsequent scratching is modulated by pain-mediated neurological pathways. Therefore, itching at night and subsequent scratching may be the basis of sleep disorders in individuals with eczema [41]. Asthma and wheezing symptoms were positively associated with self-reported sleep problems in this study. Indeed, asthma is a causal factor in sleep disorders [8]. Mechanistically, this may involve worsening of symptoms, including increased bronchial hyperresponsiveness and changes in inflammatory pathways.

A main advantage of our study was the large sample of adults over the age of 20 years old in the United States. Further, the relationship between sleep disorders and allergy-related outcomes was not altered after fully adjusting for confounding factors. The associations between various sleep disorders and allergies were analysed individually, thereby increasing accuracy of the analysis. Nevertheless, our study has several limitations. A main limitation is the cross-sectional design, which precludes determination of causal relationships. The other is the definition of sleep disorder. For the assessment of sleep disorders, the NHANES database only provides self-questionnaires about sleep disorders. Clinical studies use more powerful representations to define sleep disorders, such as PSG [39].We refer to the research of Scinicariello et al., and use the dataset of the NHANES database to define sleep disorders [9]. However, since the questionnaire is subject to subjective assessment, this inevitably biases the results.

In conclusion, this study used an epidemiological investigation to evaluate the relationship between sleep disorders and allergy-related outcomes in US populations. Based on NHANES data representing adults aged > 20 years in the US, sIgE sensitisation is positively correlated with sleep duration. OSA symptoms, daytime sleepiness, and sleep problems were positively correlated with various self-reported allergic conditions. Collectively, these results demonstrate a positive relationship between sleep disorders and allergy-related outcomes. Future studies investigating the mechanisms underlying this relationship are warranted.

## Data Availability

The datasets used and/or analysed during the current study are available from the corresponding author on reasonable request.
